# The regional differences and influencing factors of tourism development on Hainan Island, China

**DOI:** 10.1371/journal.pone.0258407

**Published:** 2021-10-08

**Authors:** Shengrui Zhang, Hongrun Ju

**Affiliations:** 1 Management College of Ocean University of China, Qingdao, China; 2 School of Tourism and Geography Science, Qingdao University, Qingdao, China; Northeastern University (Shenyang China), CHINA

## Abstract

Exploring the spatial pattern of tourism resources and tourism economy is vital to improve the utilization efficiency of tourism resources and promote sustainable tourism development. This research investigated the quantity and types of tourism resources and analyzed the spatial patterns of tourism resources on Hainan Island from the perspectives of spatial variation and spatial association. The spatial and temporal pattern of the number of tourists and tourism revenue during 2010–2019 were further analyzed. The influencing factors of tourism development were explored based on the geographic detector. The results showed that 10425 tourism resources exist on Hainan Island, and the type of buildings and facilities had the largest number of tourism resources. The geological landscape, astronomical phenomena and meteorological landscapes, buildings and facilities, ruins and remains, tourism commodities, and human activities showed significant spatial agglomeration. Domestic tourism was far more developed than inbound tourism in terms of the number of tourists and tourism revenue. However, the spatial difference of tourism resources and tourism economy was apparent on Hainan Island. Factor analysis showed that the quantity of hotels, the proportion of tertiary industry in the GDP, and the regional population were the most influential factors for the distribution of tourism resources, while the density of the road network, the quantity of hotels, the per capita GDP, the proportion of tertiary industry in GDP, the regional population, and the quantity of tourism resources showed obvious influences on the tourism economy of Hainan Island. Interactions of the factors mainly fell into three types: synergistic increases, single factor weakening, and nonlinear weakening. It is suggested that the local government should fully exploit diversity types of tourism resources on Hainan Island to attract more tourists and improve the tourism revenue; improving the inbound tourism, and to strengthen the construction of road network on Hainan Island.

## Introduction

As the largest island completely located in the tropical region of China, Hainan has rich tourism resources, unique cultural customs, and favorable conditions for the development of tourism [1]. Hainan’s spatial advantages and favorable policies have provided opportunities for the high-quality development of tourism in the area. Since the implementation of the international tourism island strategy in 2009, Hainan’s tourism industry had achieved rapid development. By 2019, Hainan’s tourism revenue accounted for about 19.92% of the area’s GDP—much higher than the global average (6.61%) and average level in China (11.05%) [2]. Notably, the offshore duty-free policy greatly promoted the development of domestic tourism. In 2020, the outbreak of COVID-19 limited the development of outbound tourism, which further boosted domestic tourism development in China, indicating that the continued growth trend of Hainan’s tourism would not change in the new era [3]. On the other hand, there has been a homogenized development trend in Hainan’s tourism, which could easily lead to vicious competition and threaten the coordinated development of Hainan’s tourism [4]. In the relevant studies on Hainan, scholars have focused on the development of tourism resources [5, 6], tourism management [7–9], problems in the development of the tourism industry and countermeasures [10–12], impacts of tourism development on local environment and economy [13–16].

Island tourism refers to the phenomenon of the development of tourism on an island that advances the establishment of family guesthouses, corporate hotels, and other related commercial areas, as well as an integration of the island’s scenic spots and urban developments [17]. Tourism resources of island were special with its natural and human attractions, such as coral reefs, unique flora and fauna and minority culture. Tourism resource evaluation is a hot spot in island tourism research. Priskin constructed a natural resource evaluation index system including 29 indicators from four aspects: attractiveness, accessibility, infrastructure status, and environmental degradation, and evaluated the natural resources of island in central Western Australia [18]. Li et al. built an evaluation system of 26 indicators including resource value, development status, location, transportation, environmental capacity, and economic benefits, and applied the analytic hierarchy process and projection tracking model to evaluate the development potential of tourism resources for island counties in China [19]. Some scholars also focused on the spatial structure of the tourism resources on island. Yang et al. studied the junction point, path and domain constituting tourism spatial structure of island destination and propose optimal suggestion for the tourism spatial structure [20]. Shen and Tian took 41 high quality tourism attractions in Hainan island as an example, analyzed the spatial structure and its evolution based on nearest neighbor index and geographic concentration index [6]. However, due to the difficulties of acquiring exhaustive data of tourism resources on island, studies on the regional differences of tourism resources on tourism islands were still lack.

Tourism revenue and the number of tourists were important topic related to tourism development. Jang et al. estimated the lost tourism revenue in Geoje Island from the 2011 marine debris pollution event in South Korea base on the analysis of visitor count [21]. Kristiana et al. found that the improving of quality of facilities and services at tourism destination could help increase the number of tourists [22]. Joshi et al. found that the international tourism revenue was more responsive to policies and regulations favoring tourism, abundance of natural resources, richness in cultural heritage, and health and hygiene than they are to infrastructure, safety, price competitiveness, and other variables [23]. Chen et al. pointed out that climatic seasonal factors have significant pulling and pushing effects on seasonal patterns of tourism demand, with temperature being the main factor [24]. However, comprehensive and quantitative studies on the influencing factors of the natural, social, and economic aspects on the tourism development remained insufficient. It would be helpful to measure different factors’ impacts on the tourism resources, tourism revenue and the number of tourists. Unlike previous studies mostly explored the drivers from the perspective of the time dynamic, this study would analyze the influencing factors from the perspective of spatial difference based on a novel method named geographical detectors.

Overall, this paper will comprehensively describe the tourism development pattern including tourism resources, tourism revenue and number of tourists of Hainan Island and identifying the influencing factors. First, the structural types and quantitative characteristics of tourism resources on Hainan Island will be analyzed and the spatial pattern of tourism resources at township level will be described. Then, this paper will analyze the development of Hainan’s tourism economy from two perspectives of the quantity of tourists and tourism revenue in 2010–2019. Finally, the influencing factors and the influencing mechanisms of Hainan’s tourism development will be analyzed. Optimization measures will be proposed to improve the efficiency of tourism resources exploitation and realize sustainable tourism development on Hainan Island.

## Data and methods

### Data source

**Fig 1:**
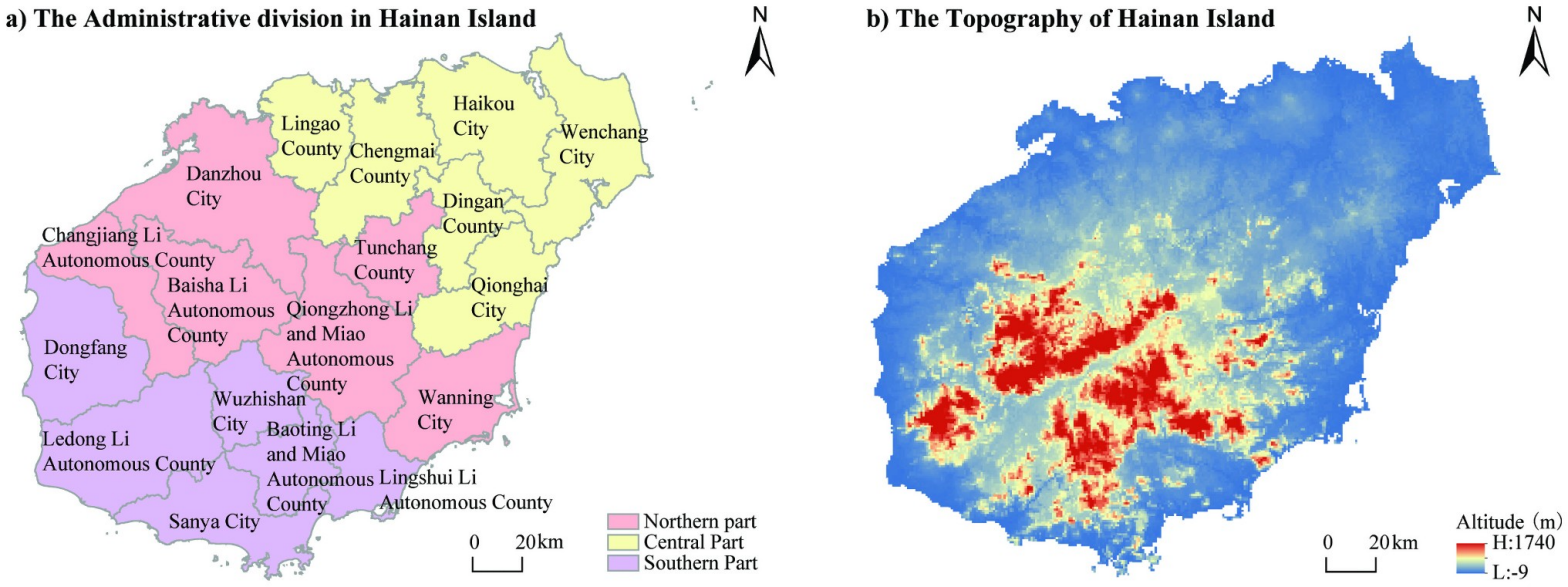
Administrative district and topography of Hainan Island.

The data include tourism resources, tourism economic data, and DEM data. The divisions of prefectural and county units were based on the administrative divisions of 2018 to avoid data inconsistencies caused by adjustments to the administrative divisions over time (Fig 1a). Due to the relatively small number of tourists and the lack of relevant data, we did not analyze the situation in Sansha City. In addition, since the Yangpu area had not yet established an independent tourism administration department at the time of this study, the area’s relevant data were incorporated into Danzhou City.

The tourism resource data were acquired from four sources: academic documents, the tourism planning of the counties, the official travel websites of the counties, and field surveys carried out in 2018. The attributes and spatial coordinates of tourism resources were obtained via field research, which covered 18 counties and cities on Hainan Island.

The tourism economic data were acquired from Hainan Statistical Yearbook (2010–2020) and China Statistical Yearbook (2010–2020). According to the classification of tourism economic indicators in the statistical yearbook, we selected two indicators, the quantity of tourists and tourism revenue, to describe the development of Hainan’s tourism economy. The DEM data were obtained from the National Basic Geographic Information System database at a resolution of 500 m (Fig 1b).

### Methodology

This paper analyzes the spatial pattern of tourism development on Hainan Island from three perspectives: spatial differences, spatial autocorrelation, and spatial agglomeration effects. The spatial differences are expressed as the coefficient of variation (*CV*), while the global and local Moran’s *I* indexes are used to test the global and local spatial associations of tourism development. The influence of each factor is measured using the Geographic Detector.

#### Spatial differences

The coefficient of variation (*CV*) was used to describe the spatial differences in tourism resources, which were calculated as follows [25, 26]:

$$SD=\sqrt{\;\frac{\text{1}}{n}\sum_{i=1}^{n}{{(X}_{\text{i}}–\bar{X}\text{)}}^{\text{2}}}$$ (1)

$$CV=SD/\bar{X}$$ (2)

where *CV* refers to the coefficient of variation, *SD* is the standard variance, *n* is the number of units at different scales, *X*_*i*_ refers to the number of the objects of tourism resources in the *i*th administrative unit (i = 1, 2…, n.), and $\bar{X}$ refers to the average objects of tourism resources in each administrative unit. Here, the larger the *CV* is, the greater the spatial variation of tourism resources will be.

#### Spatial association indicators

(1) Global indicators of spatial association
  In this paper, Moran’s index was adopted to conduct the global autocorrelation analysis of tourism resources, and the global correlation of tourism resources was calculated using the following calculation formula [27]:
$$\text{Global}\;\text{Moran}’\text{s}\;I=\;\frac{n}{\sum_{i=1}^{n}\sum_{j=1}^{n}w_{ij}}\frac{\sum_{i=1}^{n}\sum_{j=1}^{n}w_{ij}(X_{i}–\bar{X})(X_{j}–\bar{X})}{\sum_{i=1}^{n}{\left(X_{i}–\bar{X}\right)}^{\text{2}}}$$ (3)
  where *n* is the number of administrative areas, *X*_*i*_ and *X*_*j*_ are the number of tourism resources in *i* administrative unit and *j* administrative unit, $\bar{X}$ is the average, and *w*_*ij*_ is the spatial weight. The values of the global Moran’s *I* range from −1 to +1. Positive values indicate the clustering of similar values across geographic space. In contrast, negative values indicate that the neighboring values are more dissimilar than expected by chance.
(2) Local indicators of spatial association (LISA)
  The local autocorrelation coefficient (LISA) was used to express the local agglomeration of tourism resources, including high–high agglomeration, low–low agglomeration, high–low agglomeration, and low–high agglomeration [27]:
$$\text{Local}\;\text{Moran}’\text{s}\;I=\;\frac{X_{i}–\bar{X}}{S_{i}^{\text{2}}}\underset{j=1,\;j\neq \text{i}}{\sum^{n}}w_{ij}\text{(}X_{j}–\bar{X}\text{)}$$ (4)
  where $S_{\text{i}}^{\text{2}}$ refers to the variance of tourism resources, and other variables are consistent with those described in (3). A positive Local Moran’s *I* value indicates high–high or low–low agglomeration, which means that the quantity of tourist resources is similar in two adjacent administrative units. A negative Local Moran’s *I* value indicates high–low or low–high agglomeration, which means that the quantity of tourism resources is very different in the adjacent administrative unit.

#### Geo-detector

The factor detector was used to analyze the relative influence of various factors on the tourism development of Hainan and the interaction detector was used to analyze the interaction mechanisms among each factor. The influencing indicators of tourism development was selected based on three perspectives: natural conditions, social environment, and economic development. The natural indicators included relief amplitude and average elevation. The social environment included the quantity of residents, minority populations, road network density, and quantity of hotels. The economic development indicators included the per capita gross domestic product and the proportion of tertiary industry in GDP.

(1) Factor detector
  Factor detectors explore the potential factors or explanatory variables from the perspective of spatial heterogeneity and can be used to quantitatively determine the relative influence of all possible influencing factors [28]. The calculation formula is as follows:
$$p=1-\frac{\sum_{i=1}^{N}n_{i}\sigma_{i}{}^{2}}{n\sigma^{2}}$$ (5)
  where *p* is the influence of one factor; *n* is the stratification of variable *Y* or factor *X*; *n*_*i*_ and *n* are the number of units in layer *i* and the whole region, respectively; and $\sigma_{i}{}^{2}$ and *σ*^2^ are the variance of the *Y* values. If factor *x* completely affects the development of tourism, then *p* tends to be 1. If factor *x* has nothing to do with the tourism development data, then *p* tends to be 0. The range of the *p* value is [0,1], where a greater value of *p* indicates a greater influence of the factor.
(2) Interaction detector
  The interaction detector is mainly used to identify whether different influencing factors exert interaction influences on tourism development. If the results show that an interaction influence exists, then this method can identify the type of interaction and reflect whether the combined effect of the two factors will increase or weaken the factor’s explanatory power for tourism development. In this study, the interaction detector quantitatively revealed the interactions of two possible influencing factors, as shown below [28]:
$$\text{Nonlinear}\;\text{weakening}:\;p(M\cap N)<\text{Min}\left(p\left(M\right),p\left(N\right)\right)$$
$$\text{One-factor}\;\text{weakening}:\;\text{Min}\left(p\left(M\right),p\left(N\right)\right)<p(M\cap N)<\text{Max}\left(p\left(M\right),p\left(N\right)\right)$$
$$\text{Mutually}\;\text{reinforcing}:\;\text{Max}\left(p\left(M\right),p\left(N\right)\right)<p(M\cap N)<\left(p\left(M\right)+p\left(N\right)\right)$$
$$\text{Independent}:\;p(M\cap N)=p\left(M\right)+p\left(N\right)$$
$$\text{Nonlinear}\;\text{enhancement}:\;p(M\cap N)>\left(p\left(M\right)+p\left(N\right)\right)$$

In the ArcGIS platform, two layers of impact factors (such as *M* and *N*) can be superimposed to form layer *O*. Then, the *P* values of *M*, *N*, and *O* can be calculated and substituted into the above formula to determine whether the two factors feature interactions and whether these interaction are enhanced or weakened.

The factor analysis and interaction analysis of the influencing factors in this study were all based on the *GD* package in the *R* language, which can automatically determine the best discretization method and amount of discretization for each factor. For the discretization of all factors in this study, the equidistant method, natural discontinuous point method, and quartile method were adopted, and the discretization quantity was selected from the 4–6 discretization divisions with the greatest influence.

## Results

### Numerical features of tourism resources

The results indicated a total of 10425 tourism resource objects on Hainan Island, while the numbers of different types of tourism resources were significantly different. According to the National Standard for the “Classification, investigation and evaluation of tourism resources” (GB/T18972-2017), the tourism resources were divided into 8 main types, 23 sub-types, and 110 fundamental types. In the case of Hainan Island, Buildings and Facilities was the largest main type, and included 4807 tourism resource objects, followed by Geological Landscapes, Water Landscapes, and Ruins and Remains with more than 1000 objects. By contrast, the tourism resource objects of Tourism Commodities, Human Activities, Biological Landscapes, and Astronomical Phenomena and Meteorological Landscapes accounted for about 10% of the total. In terms of subtypes, practical buildings and facilities, cultural landscape complexes, natural landscape complexes, and lakes were the major types of the tourism resources. However, the Industrial products, Astronomical landscapes, and Natural markers and natural phenomena were relatively rare in Hainan Island (Table 1).

**Table 1:** The types and quantity of tourism resources on Hainan Island.

Main Types			Subtype		
The types	Number	Proportion (%)	The types	Number	Number of the fundamental types
A Geological Landscapes	1839	17.64	AA Natural Landscape Complex	1673	17
			AB Geological and tectonic features	37	
			AC Surface morphology	116	
			AD Natural markers and natural phenomena	13	
B Water Landscapes	1600	15.35	BA Rivers	242	11
			BB Lakes	1111	
			BC Groundwater	47	
			BE Sea surface	200	
**C** Biological Landscapes	254	2.43	CA Vegetation landscape	206	8
			CB Wildlife habitat	48	
D Astronomical Phenomena and Meteorological Landscapes	33	0.32	DA Astronomical landscape	12	4
			DB Weather and climate phenomena	21	
E Buildings and Facilities	4807	46.11	EA Cultural landscape complex	1767	40
			EB Practical buildings and facilities	2256	
			EC Landscape architecture	784	
F Ruins and Remains	1085	10.41	FA Material cultural remains	811	10
			FB Intangible cultural remains	274	
G Tourism Commodities	536	5.14	GA Agricultural products	466	14
			GB Industrial products	4	
			GC Hand-made Crafts	66	
H Human Activities	271	2.60	HA Personnel activity record	119	5
			HB Festivals	152	
**Sum**	10425	100	22	10425	109

**Fig 2:**
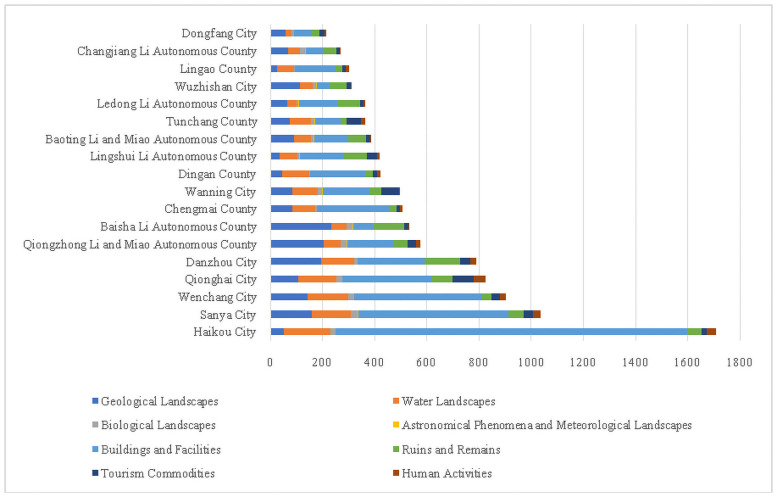
The quantities and structural of tourism resources at a regional scale on Hainan Island.

The distribution of tourism resources was unevenly in Hainan Island. Haikou City, Sanya City, and Wenchang City had the largest number of tourism resources, including 1707, 1037, and 904 objects, respectively. By contrast, the number of tourism resource objects in Lingao County, Changjiang Li Autonomous County, and Dongfang City were only 303, 269, and 213, respectively (Fig 2). The structure of the different types of the tourism resources were various over space. Buildings and Facilities was the major type for most cities and counties, including Haikou City, Sanya City, Wenchang City, Qionghai City, Chengmai County, Dingan County, Lingao County. Some areas were dominated by Buildings and Facilities and Geological Landscapes, including Baoting Li and Miao Autonomous County, Dongfang City and Qiongzhong Li and Miao Autonomous County. By contrast, Wuzhishan City, Tunchang County, Changjiang Li Autonomous County, and Danzhou City had a more balanced structure with multiple types such as Geological Landscapes, Buildings and Facilities, Water Landscapes, as well as Ruins and Remains. Only Baisha Li Autonomous County was dominated by Geological Landscapes tourism types.

### Spatial differences and spatial autocorrelation of tourism resources at the township level on Hainan Island

Influenced by the natural and cultural environment, the quantity of tourism resources varies greatly at different scales on Hainan Island. The overall coefficient of variation was 61.26% of Hainan Island. At the township level, the *CV*s of the Astronomical Phenomena and Meteorological Landscapes, Buildings and Facilities, and Human Activities were all higher than 80% (Table 2), indicating that the spatial differences between these tourism resources are significant. Astronomical Phenomena and Meteorological Landscapes were mainly concentrated in Changjiang Li Autonomous County, Qiongzhong Li and Miao Autonomous County, Baoting Li and Miao Autonomous County, and Wuzhishan City, accounting for about 51.51% of the total. Buildings and Facilities were mainly concentrated in Haikou, Sanya, and Wenchang, accounting for about 50.09% of the total. Human Activities were mainly concentrated in Qionghai, Haikou, and Sanya, accounting for about 40.96% of the total.

**Table 2:** The *CV*s and Moran’s *I* values of tourism resources on Hainan Island.

Indicators	SUM	Geological Landscapes	Water Landscapes	Biological Landscapes	Astronomical Phenomena and Meteorological Landscapes	Buildings and Facilities	Ruins and Remains	Tourism Commodities	Human Activities
*CV* (%)	61.26	58.21	49.51	51.96	89.54	110.99	51.88	65.49	80.88
Moran’s *I*	0.2013	0.2013	0.2013	0.2013	0.2013	0.2013	0.8810	0.2013	0.2013
Z score	0.1629	2.3504	1.5815	-0.8446	3.5451	3.4867	2.2733	5.9635	7.523
P value	0.8705	0.0187**	0.1137	0.3983	0.0004**	0.0005**	0.0230**	0.0000**	0.0000**

** indicates that the value is significant at a 0.01 level.

**Fig 3:**
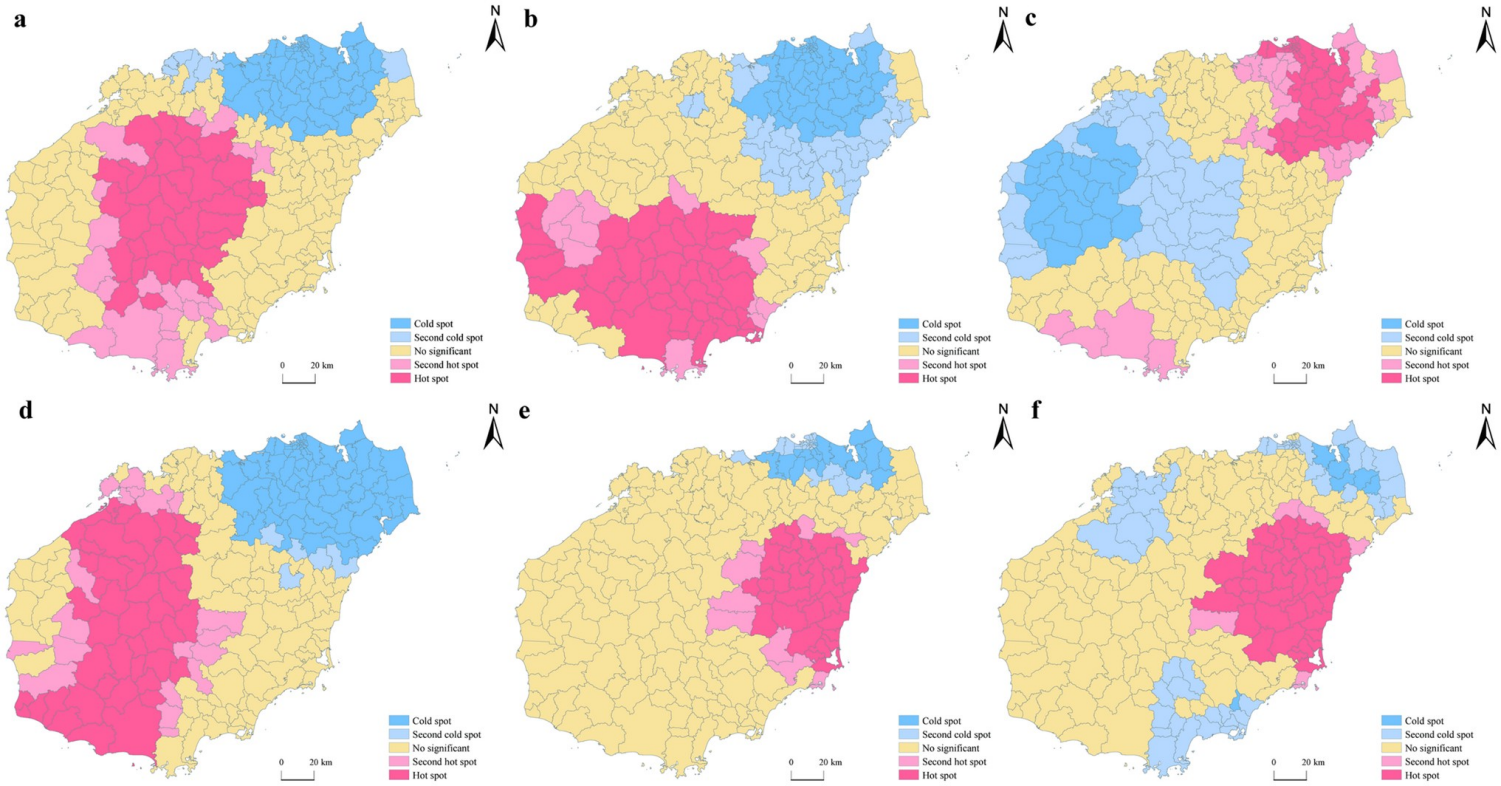
The hot/cold spatial patterns of tourism resources in Hainan (a: Geological Landscape; b: Astronomical Phenomena and Meteorological Landscapes; c: Buildings and Facilities; d: Ruins and Remains; e: Tourist Commodities, f: Human Activities).

The Global Moran’s *I* values of Geological Landscapes, Astronomical Phenomena and Meteorological Landscapes, Buildings and Facilities, Ruins and Remains, Tourism Commodities, and Human Activities were all significant at a level of 0.01 (Table 2), which indicates that these six types of border tourism resources all feature obvious spatial agglomeration phenomena at the township scale. Conversely, the Global Moran’s *I* of the total amount of tourism resources, Water Landscapes, and Biological Landscapes were not significant (Table 2), indicating a relatively random distribution of all tourism resources and these two types of tourism resources at the township scale. Therefore, local autocorrelation analysis was only applied to Geological Landscapes, Astronomical Phenomena and Meteorological Landscapes, Buildings and Facilities, Ruins and Remains, Tourism Commodities, and Human Activities (Fig 3).

The hot/cold spatial patterns of Geological Landscapes, Water Landscapes, and Ruins and Remains were similar. The hot spots were concentrated in the southern region of Hainan Island. The cold spots were mainly concentrated in the northeast Hainan Island, and Haikou City and Chengmai County were the lowest density areas (Fig 3a, 3b and 3d). By contrast, the types of Tourism Commodities and Human Activity were mainly concentrated in the central and eastern parts of Hainan Island. High-high clusters were mainly distributed in the towns of Tayang, Boao, Tanmen, etc. The cold spots of Tourism Commodities were mainly concentrated in the northern towns of Haikou City, Wenchang City, Chengmai County, and Lingao County (Fig 3e), while the cold spots of Human Activity were located in the north of Haikou City and Wenchang City, in the northwest of Danzhou City, and in the southeast of Sanya City and Lingshui County, including Bolian Town, Dongge Town, Penglai Town, and Leiming Town, which all had low distribution density (Fig 3f).

The spatial pattern of hot/cold spots of Buildings and Facilities were different from the other types of tourism resources. The hot spots was concentrated in the northeast and the south of the island. At the township level, Longhua District, Qiongshan District, Meilan District, Xiuying District, Huiwen Town of Wenchang City, Boao Town of Qionghai City, and Yazhou District of Sanya City all showed high–high clusters. The cold spots were mainly concentrated in the central and western regions of Hainan Island, including Maodao Town, Changhao Town, Yaxing Town, Wangxia Town, Jiangbian Township, and Donghe Town (Fig 3c).

### Spatiotemporel pattern of the tourism economy on Hainan Island from 2010 to 2019

Generally, the average number of tourists in Hainan totaled 51.41 million per year during 2010–2019, with a cumulative growth rate of 15.70%. In 2013–2014, the number of tourists in Hainan experienced the highest growth rate (30.40%). During 2010 to 2019, the average tourism revenue of the island reached 9.21 billion dollars, with a cumulative growth rate of 19.31%; the highest growth rate, 20.82%, was recorded in 2016–2017.

**Fig 4:**
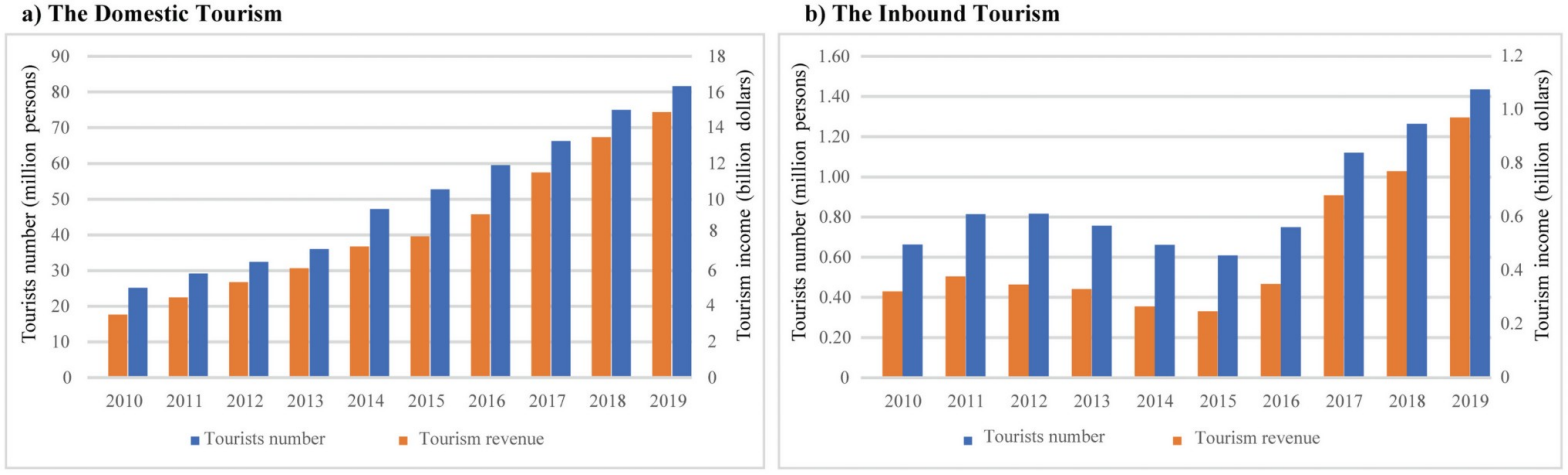
The temporal changes of (a) domestic tourism and (b) inbound tourism economy in 2010–2019 on Hainan Island.

The development of the tourism economy on Hainan Island has been dominated by domestic tourism, with inbound tourism as a supplement. In terms of domestic tourism, the average number of tourists on the island reached 50.56 million per year in the past decade, with a cumulative growth rate of 15.83%. The highest growth rate occurred in 2013–2014, up to 31.31%. During this period, the average tourism revenue of all counties and cities in Hainan reached 8.63 billion dollars, with a cumulative growth rate of 19.68%; highest growth rate of 25.64% occurred in 2016–2017 (Fig 4a).

In terms of inbound tourism, the average number of tourism on Hainan Island during the 10 years was 0.89 million per year, with a cumulative growth rate of 10.14%. The cumulative growth rate of inbound tourism revenue was 14.81%, which is slightly higher than the national average (14.06%). However, the development of inbound tourism on Hainan Island experienced an obvious fluctuation during the ten years. In 2010–2012, the average annual growth rate of inbound tourism was 23.01%, and the average annual growth rate of inbound tourism revenue was 8.08%. During 2013–2015, the inbound number of tourists and tourism revenue decreased at an average annual changing rate of -19.57% and -25.08%, respectively. In 2016–2019, the inbound tourism development recovered, with an average annual growth rate of 38.46% for inbound number of tourists, and an average annual growth rate of 66.65% for inbound tourism revenue (Fig 4b).

**Fig 5:**
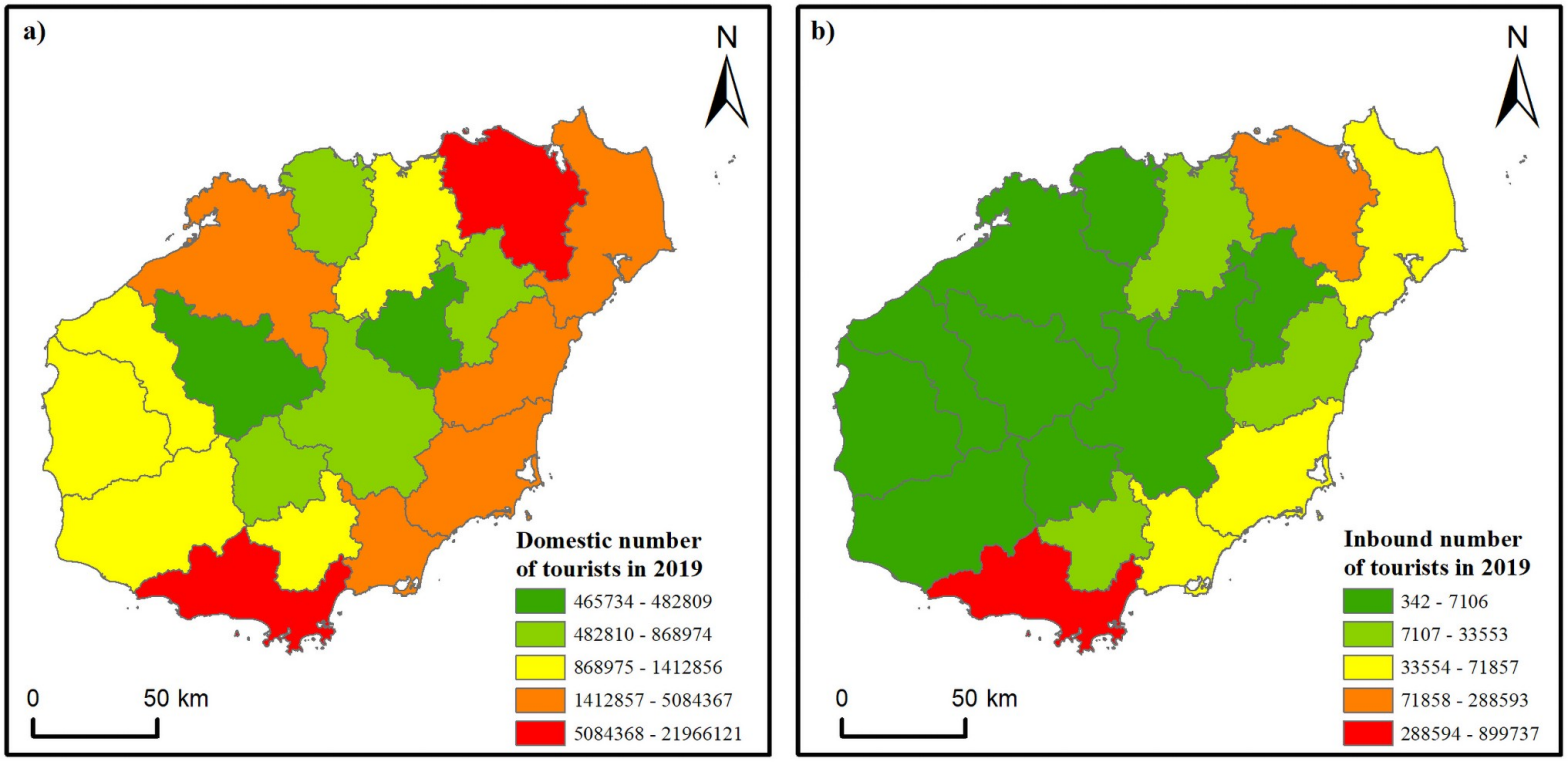
The spatial pattern of (a) domestic tourists and (b) inbound tourists in 2019 on Hainan Island.

The spatial pattern of number of tourists was present in Fig 5, and the pattern of tourism revenue was ignored because the lack of statistic data for each city and county in Hainan. It is shown that the domestic number of tourists was highest in Haikou city in the north and Sanya city in the south of Hainan Island. The eastern parts of the island also attracted a large number of domestic tourists, followed by the western parts of the island. The central parts of Hainan Island had the least tourists. By contrast, the number of inbound tourists was low for most areas on Hainan Island, and the eastern parts of Hainan Island along the sea attracted more tourists. Sanya and Haikou cities were most popular for international tourists.

By comparing the spatial patterns of tourism resources and number of tourists (Figs 4 and 5), an apparent spatial difference could be found between them. The spatial pattern of domestic number of tourists was most similar to that of the tourism resource type of Buildings and Facilities, where was high in the northeastern and southwestern parts and low in the western and central parts of the island. However, the other types of tourism resources such as Geological Landscape, Astronomical Phenomena and Meteorological Landscapes, Ruins and Remains, Tourist Commodities, and Human Activities all had an obvious spatial dislocation phenomenon with the distribution of domestic tourists and inbound tourists. This phenomenon may indicate an insufficient exploitation of these types of tourism resources.

## The influence and interaction of factors on tourism development on Hainan Island

Geographical detector was used to analyze the influence of factors on tourism resources and number of tourists. The influence on tourism revenue was not processed due to the lack of spatial distribution data of the tourism revenue. Three factors showed significant influence on the tourism resources: the quantity of hotels, the proportion of tertiary industry in GDP, and regional population (Table 3). The quantity of hotels had the greatest explanatory power (0.7707), indicating that the spatial patterns of tourism resources were consistent with the distribution of hotels. The main reason for this result is that quantity of hotels and the number of tourism resources can objectively reflect regional tourism reception capacity, giving the two factors a strong correlation. The second influential factor was the proportion of tertiary industry in GDP, whose explanatory power reached 0.7383. It was because tertiary industry drove the development of regional tourism resources. The explanatory power of population scale was also high (0.7051), mainly because an increase in population size correlated with an increase in the tourist market, indicating a higher demand for tourism resources development.

**Table 3:** Relative influence of the factors on tourism resources and the number of tourists on Hainan Island.

Influencing criteria	Influencing factors	q value for tourism resource	q value for number of tourists
V_1_ Natural conditions	V_11_ relief amplitude	0.3825	0.2736
	V_12_ average elevation	0.5548	0.3230
V_2_ Social conditions	V_21_ regional population	0.7051**	0.6548*
	V_22_ ethnic minority population	0.2763	0.2933
	V_23_road network density	0.6543	0.9456**
	V_24_ quantity of hotels	0.7707**	0.9651**
V_3_ Economic conditions	V_31_ per capita gross domestic product	0.6604	0.9484**
	V_32_ the proportion of tertiary industry in GDP	0.7383**	0.9369**
V_4_Tourism resources	V_41_ The quantity of tourism resources	--	0.6326*

** indicate the factors were significant at a level of 0.05, and

* indicate the factors were significant at a level of 0.1.

Six factors had significant influence on the number of tourists. Road network density, quantity of hotels, per capita gross domestic product, and the proportion of tertiary industry in GDP were significant at a level of 0.05, while population and the quantity of tourism resources were significant at a level of 0.1 (Table 3). Notably, the explanatory power of quantity of hotels reached 0.9651, mainly because the development of hotels (the main reception facilities) was closely related to the choices of tourists. The explanatory power of per capita gross domestic product was 0.9484, mainly because the higher the per capita GDP is, the greater the residents’ abilities to travel and the larger the tourism market will be. In addition, road network density had a high influence because higher density of the road network could greatly increase the accessibility of tourism resources. The proportion of tertiary industry in GDP also had a significant impact, because a larger proportion of tertiary industry will lead to greater development of the local service industry and higher-quality service facilities. Moreover, a greater number of tourism resources in an area means that more tourists will be attracted to that area, thus contributed to the increase of tourists.

**Fig 6:**
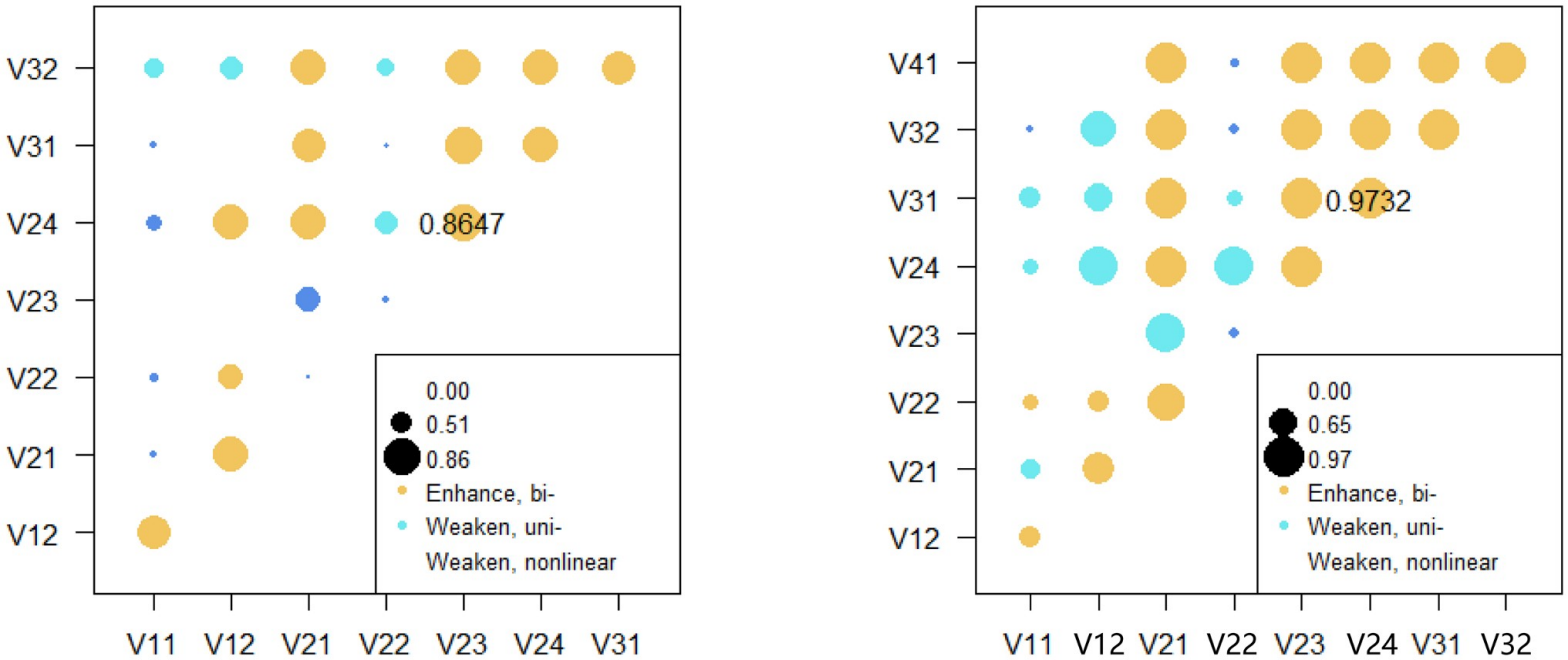
The interactions of factors on the spatial pattern of (a) tourism resources and (b) number of tourists on Hainan Island.

The interactions of the influencing factors on tourism resources showed that the interaction effects of natural conditions, social environment, and economic development mainly fell into three types: synergistic increases, single factor weakening, and nonlinear weakening (Fig 6a). The interaction between road network density and quantity of hotels had the largest influence on the spatial patterns of tourism resources, reaching 0.8647. The interaction between natural factors was synergistically enhance, while the interactions with social and economic factors presented various types. In particular, the interactions between relief amplitude and other social and economic factors were weakening, while the interactions between average elevation and socioeconomic factors were mostly synergistically enhance. Various types of interactions were observed among the social factors. For example, the interaction between the quantity of hotels and the population and the interaction between road network density and quantity of hotels were all synergistically enhanced. However, interaction between ethnic minority population and other factors were mostly weakening. The interactions of social factors and economic factors, interactions between economic factors were mainly synergistic enhance.

The interactions of the influencing factors on the number of tourists were mainly synergistical increasing and single-factor weakening; only part of the interaction presented nonlinear weakening (Fig 6b). The interaction between GDP per capita and the quantity of hotels had the largest influence on the number of tourists, reaching 0.9732. The interactions between natural factors showed synergistic enhancement, while the interactions between natural factors and social factors or economic factors were mainly single-factor weakening. In addition, the interactions between the social and economic factors or tourism resources were mainly synergistically enhanced. Only the interactions between the size of the ethnic minority population and some economic development factors and tourism resources were weakened.

## Conclusions

In this study, we established a comprehensive database of tourism resources, number of tourists and tourism revenue on Hainan Island during 2010–2019. Based on the dataset, the spatial patterns of tourism resources and the tourism economy of Hainan Island were analyzed. The influencing factors and their interactions were further analyzed based on geographical detector.

The main conclusions of this paper are as follows: (1) 10425 tourism resource objects was investigated on Hainan Island. Buildings and Facilities was the largest main type, and Practical Buildings and Facilities was the largest sub-type. Haikou City and Dongfang City were the county-level units with the largest and smallest numbers of tourism resources, respectively; (2) The spatial differences in Hainan’s tourism resources were significant, and the *CV*s of the Astronomical Phenomena and Meteorological Landscapes, Buildings and Facilities, and Human Activities were all higher than 80%. Geological Landscapes, Astronomical Phenomena and Meteorological Landscapes, and Ruins and Remains all showed high–high clusters in the southwest and low–low clusters in the northeast of the island, respectively. Buildings and Facilities were concentrated in the northeast and southwest of the island and dispersed in the central area. Tourism Commodities and Human Activities were gathered in the central and eastern areas and dispersed in other areas; (3) During 2010–2019, the cumulative growth rates for the number of tourists and tourism revenue on the island were 15.70% and 19.31%, respectively. The domestic tourism was far more developed than the inbound tourism based on the data of tourism revenue and tourists. In addition, the spatial difference between tourism resources and tourism economy was apparent on Hainan Island; (4) The quantity of hotels, proportion of tertiary industry in GDP, and regional population had a significant impact on the spatial patterns of tourism resources on Hainan Island, while the density of the road network, the number of hotels, the per capita GDP, the proportion of tertiary industry in GDP, the regional population, and the quantity of tourism resources were found to had significantly effects on the spatial patterns of the number of tourists on Hainan Island. The interactions of factors of tourism resources mainly fell into three types: synergistic increases, single factor weakening, and nonlinear weakening, while the interactions of the number of tourists were mainly synergistical increasing and single-factor weakening.

Based on the above results, three suggestions were proposed for the sustainable development of tourism on Hainan Island. First, the tourism resource and their attractions for tourists showed an obvious spatial dislocation phenomenon. It indicates that the tourism resources on Hainan Island were not sufficiently developed. The local government should take advantages of the diversity of different types of tourism resources on Hainan Island to attract more tourists and improve the tourism revenue. Second, the development of inbound tourism on Hainan Island is less developed compared with the domestic tourism. It is wise to learn from international famous tourism islands such as the Hawaii to create an international tourist destination. Third, according to the factor analysis, the density of road network would greatly promote the growth of the tourism market, and could result a synergistic enhance effects when interact with other factors. Thus, the government should strengthen the construction of basic road network on Hainan Island.

Due to the lack of county-level tourism revenue data, we only analyzed the spatial pattern of the quantity of tourists. In the future, the spatial patterns of the differences between tourism resources and tourism economy deserved further quantitative exploration. Also, the evaluation of the tourism resources could be done in the future with multi-sources such as social network.
